# Paraoxonase-1 Is a Pivotal Regulator Responsible for Suppressing Allergic Airway Inflammation Through Adipose Stem Cell-Derived Extracellular Vesicles

**DOI:** 10.3390/ijms252312756

**Published:** 2024-11-27

**Authors:** Jae Hoon Jung, Shin Ae Kang, Ji-Hwan Park, Sung-Dong Kim, Hak Sun Yu, Sue Jean Mun, Kyu-Sup Cho

**Affiliations:** 1 Department of Otorhinolaryngology, Pusan National University School of Medicine, Busan 50612, Republic of Korea; narcissism20@naver.com; 2 Department of Environmental Medical Biology, Catholic Kwandong University College of Medicine, Gangneung 25601, Republic of Korea; f2s4u@pusan.ac.kr; 3 Department of Otorhinolaryngology and Biomedical Research Institute, Pusan National University School of Medicine, Pusan National University Hospital, Busan 50612, Republic of Korea; nobleivy@naver.com (J.-H.P.); appleln@naver.com (S.-D.K.); 4 Department of Parasitology and Tropical Medicine, Pusan National University School of Medicine, Busan 50612, Republic of Korea; hsyu@pusan.ac.kr; 5 Department of Otorhinolaryngology and Research Institute for Convergence of Biomedical Science and Technology, Pusan National University Yangsan Hospital, Busan 50612, Republic of Korea; baskie23@naver.com

**Keywords:** mesenchymal stromal cells, adipose stem cells, extracellular vesicles, immunosuppression, paraoxonase-1

## Abstract

Although adipose stem cell (ASC)-derived extracellular vesicles (EVs) are as effective as ASCs in the suppression of Th2 cell-mediated eosinophilic inflammation, the role of identified pulmonary genes has not been well documented. Thus, we assessed the immunomodulatory effects of paraoxonase-1 (PON1) on allergic airway inflammation in a mouse model of asthma. Five-week-old female C57BL/6 mice were sensitized to ovalbumin (OVA) by intraperitoneal injection and challenged intranasally with OVA. To evaluate the effect of PON1 on allergic airway inflammation, the intranasal and intraperitoneal injections of recombinant mouse serum PON1 (5 μg/50 μL) were performed before the OVA challenge. We evaluated airway hyperresponsiveness (AHR), total inflammatory cells, and eosinophils in the bronchoalveolar lavage fluid (BALF), lung histology, serum immunoglobulin (Ig), cytokine profiles of BALF and lung draining lymph nodes (LLNs), the expression of interleukin (IL)-25 and transforming growth factor (TGF)-β in mouse lung epithelial cell (MLE-12 cell), and dendritic cell (DC) differentiation. The intraperitoneal and intranasal administration of PON1 significantly decreased AHR, total inflammatory cells and eosinophils in BALF, eosinophilic airway inflammation, serum total, and OVA-specific IgE. PON1 treatment, which marked reduced IL-4, IL-5, and IL-13 in the BALF and LLN but significantly increased interferon-γ and TGF-β. Furthermore, PON1 treatment significantly decreased the expression of IL-25 and increased TGF-β in MLE-12 cells. The expressions of CD40, CD80, and CD86 in immature DCs were significantly increased by PON1 treatment. The administration of PON1 ameliorated allergic airway inflammation and improved AHR through the downregulation of IL-4, IL-5, and IL-13 and upregulation of TGF-β in asthmatic mice. Furthermore, PON1 treatment decreased Th2-mediated inflammation induced by *Aspergillus* protease antigen by decreasing IL-25 and increasing TGF-β.

## 1. Introduction

Asthma is a chronic eosinophilic airway inflammation characterized by the excessive activation of type-2 helper T (Th2) cells due to the insufficient suppression of regulatory T cells (Tregs) [1,2,3,4]. Several studies have shown that mesenchymal stem cells (MSCs), including those derived from adipose tissue (ASCs), provide a significant reduction in allergic airway inflammation and reduce airway hyperresponsiveness (AHR) in experimental allergic asthma [5,6,7]. Furthermore, ASC-derived extracellular vesicles (EVs) significantly increase the expression of paraoxonase-1 (PON1), brain-expressed X-linked 2 (Bex2), insulin-like growth factor binding protein 6 (Igfbp6), and secretoglobin family 1C member 1 (Scgb1c1) in asthmatic mice, which lead to the expansion of Tregs and improvement in allergic airway diseases [8,9,10].

Paraoxonase-1 (PON1), a major anti-oxidant enzyme, has been reported to contribute to the pathogenesis of asthma [11] and many other diseases, including rheumatoid arthritis [12,13], diabetes [14], systemic lupus erythematosus [15], and psoriasis [16]. The expression and activity of PON1 in asthmatic patients were significantly lower compared to healthy controls [11,17,18]. Moreover, PON1 has been shown to decrease airway inflammation and airway remodeling in asthmatic mice and inhibit the macrophage expression of LPS-induced inflammatory cytokines and lung fibroblast proliferation [19]. Although PON1 gene expression was significantly increased in lung tissues after ASC-derived EV treatment in asthmatic mice [9], the role of PON1 in the suppression of allergic airway inflammation by ASC-derived EVs is not well documented.

The purpose of this study was to determine whether PON1 contributes to the immunomodulatory effects of ASC-derived EVs by evaluating the effects of PON1 on allergic airway inflammation, Th1-, Th2- and Treg-related cytokines in an ovalbumin (OVA)-induced asthmatic mouse model, and cytokine expression in mouse lung epithelial cells.

## 2. Results

### 2.1. AHR and Inflammatory Cells in BALF

**Figure 1:**
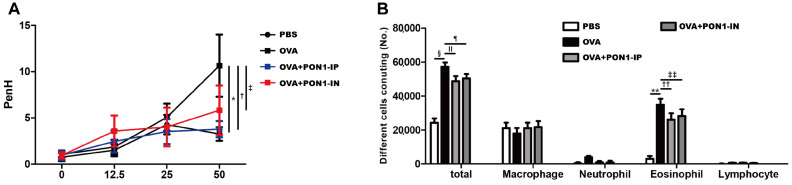
The effect of paraoxonase-1 (PON1) on airway hyperresponsiveness (AHR) and inflammatory cells in the bronchoalveolar lavage fluid (BALF). (**A**) After the methacholine challenge, AHR decreased significantly in the OVA+PON1-IP and OVA+PON1-IN groups compared to that in the OVA group. (**B**) Total inflammatory cell and eosinophil counts in BALF were significantly lowered in the OVA+PON1-IP and OVA+PON1-IN groups compared to those in the OVA group. Data are expressed as the mean ± SD of four independent experiments, each performed in triplicate. * *p* = 0.021; † *p *= 0.002; ‡ *p* = 0.049; §, ** *p* < 0.001; ǁ *p* = 0.006; ¶ *p* = 0.012; †† *p* = 0.018; ‡‡ *p* = 0.025.

The Penh values were increased with the methacholine concentration in asthmatic mice. The intraperitoneal and intranasal administration of PON1 significantly decreased AHR in asthmatic mice (*p* = 0.002 and *p* = 0.049, respectively) (Figure 1A).

The total inflammatory cell and eosinophil counts were markedly increased in the BALF of the OVA group compared to the PBS group. However, intraperitoneal and intranasal treatment with PON1 remarkably lowered the total inflammatory cell (*p* = 0.006 and *p* = 0.012, respectively) and eosinophil (*p* = 0.018 and *p* = 0.025, respectively) counts in asthmatic mice (Figure 1B).

### 2.2. Lung Histology and Inflammation Score

**Figure 2:**
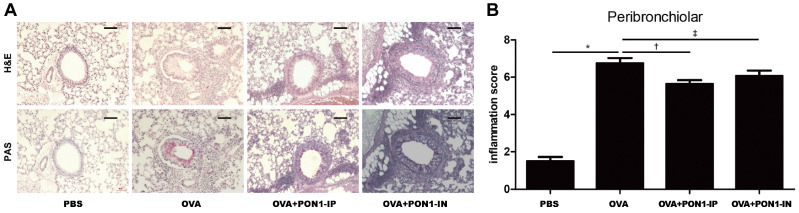
Effects of paraoxonase-1 (PON1) on lung histology and inflammation score. (**A**) Infiltration of inflammatory cells and eosinophils showed a greater decrease in the OVA+PON1-IP and OVA+PON1-IN groups than in the OVA group. The scale bar indicates 100 μm. (**B**) The inflammation score decreased significantly in the OVA+PON1-IP and OVA+PON1-IN groups compared to the OVA group around peribronchiolar areas. Data are expressed as the mean ± SD of four independent experiments, each performed in triplicate. * *p* < 0.001; † *p *= 0.003; ‡ *p* = 0.045.

High levels of eosinophil infiltration were observed around the peribronchiolar and perivascular areas in asthmatic mice. Goblet cell hyperplasia was determined by the increased number and size of goblet cells under PAS staining, and this was found in the respiratory epithelium of the OVA group. However, no clear infiltration of inflammatory cells and goblet cell hyperplasia was found in asthmatic mice treated with intraperitoneal and intranasal PON1 (Figure 2A). Furthermore, the peribronchiolar inflammation score was markedly decreased in the PON1-IP- and PON1-IN-treated group compared to the OVA group (*p* = 0.003 and *p* = 0.045, respectively) (Figure 2B).

### 2.3. Serum Total and OVA-Specific IgE, IgG1, and IgG2a Levels

**Figure 3:**
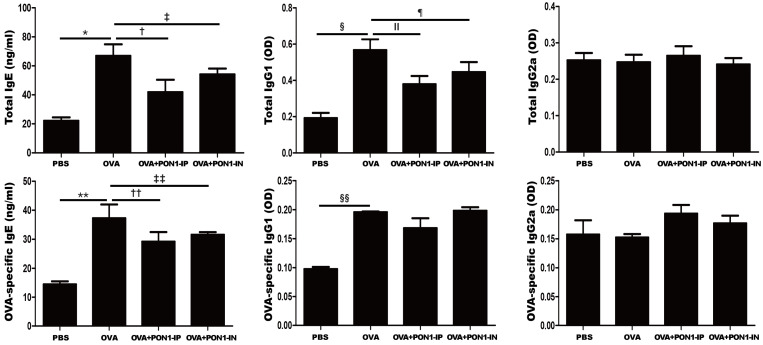
Effects of paraoxonase-1 (PON1) on serum immunoglobulin levels. Total and OVA-specific IgE and IgG1 levels were significantly increased in the OVA group compared to those in the PBS group. The intraperitoneal and intranasal administration of PON1 markedly reduced the total and OVA-specific IgE and total IgG1 levels in asthmatic mice. Data are expressed as the mean ± SD of four independent experiments, each performed in triplicate. * *p* = 0.021; † *p *= 0.028; ‡, ¶ *p* = 0.037; § *p* = 0.009; ǁ, ** *p *= 0.035; †† *p* = 0.004; ‡‡ *p* = 0.007; §§ *p* < 0.001.

The total and OVA-specific IgE (*p* = 0.021 and *p* = 0.035, respectively) and IgG1 (*p* = 0.009 and *p* < 0.001, respectively) levels were significantly higher in the OVA group than in the PBS group of asthmatic mice. However, the intraperitoneal and intranasal administration of PON1 significantly decreased the total IgE (*p* = 0.028 and *p *= 0.037, respectively), total IgG1 (*p *= 0.035 and *p *= 0.037, respectively), and OVA-specific IgE (*p *= 0.004 and *p *= 0.007, respectively) in asthmatic mice. No significant changes were observed in the total IgG2a, OVA-specific IgG1, and IgG2a levels in all groups (Figure 3).

### 2.4. Expression of Cytokines in the BALF and LLNs

**Figure 4:**
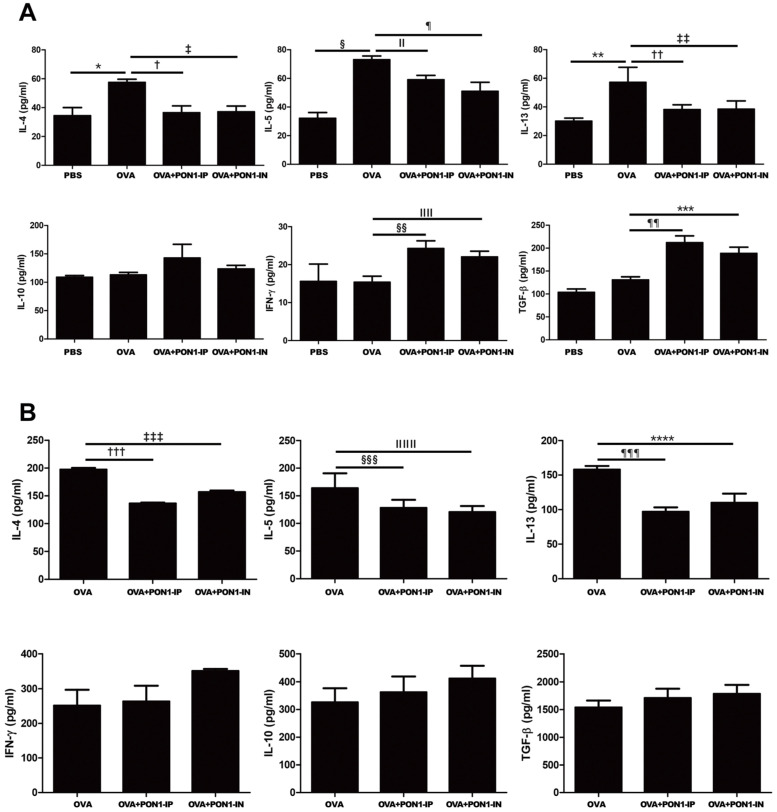
Effects of paraoxonase-1 (PON1) on cytokine levels on the bronchoalveolar lavage fluid (BALF) (**A**) and lung draining lymph nodes (LLNs) (**B**). IL-4, IL-5, and IL-13 levels were significantly increased in the BALF of the OVA group compared to those in the PBS group. Intraperitoneal and intranasal PON1 treatment remarkably reduced the levels of IL-4, IL-5, and IL-13 but increased the levels of IFN-*γ* and TGF-β in the BALF. Intraperitoneal and intranasal PON1 treatment notably decreased the levels of IL-4, IL-5, and IL-13 in the LLNs. Data are expressed as the mean ± SD of four independent experiments, each performed in triplicate. * *p* = 0.020; †, *** *p *= 0.025; ‡ *p* = 0.037; § *p* = 0.003; ǁ *p *= 0.036; ¶ *p* = 0.040; **, ‡‡, ¶¶¶ *p* = 0.047; †† *p* = 0.045; §§ *p* = 0.006; ǁǁ *p *= 0.035; ¶¶ *p* = 0.017; ††† *p* = 0.019; ‡‡‡, §§§ *p* = 0.021; ǁǁǁ *p *= 0.010; **** *p *= 0.049.

OVA-induced asthmatic mice showed a significant increase in the levels of IL-4, IL-5, and IL-13 in their BALF. However, intraperitoneal and intranasal PON1 treatment markedly decreased IL-4 (*p *= 0.025 and *p *= 0.027, respectively), IL-5 (*p *= 0.036 and *p *= 0.040, respectively), and IL-13 (*p *= 0.045 and *p *= 0.047, respectively) levels in the BALF of asthmatic mice. In contrast, intraperitoneal and intranasal PON1 treatment remarkably increased the IFN-γ level in the BALF of asthmatic mice (*p *= 0.006 and *p *= 0.035, respectively). Similarly, LLNs with intraperitoneally and intranasally administered PON1 before the OVA challenge showed considerable reductions in the levels of IL-4 (*p *= 0.019 and *p *= 0.021, respectively), IL-5 (*p *= 0.021 and *p *= 0.010, respectively), and IL-13 (*p *= 0.047 and *p *= 0.049, respectively) in asthmatic mice. Furthermore, intraperitoneal and intranasal PON1 treatment significantly increased TGF-β in the BALF of asthmatic mice (*p *= 0.017 and *p *= 0.025, respectively) (Figure 4).

### 2.5. Expression of IL-25 and TGF-β in MLE-12 Cells

**Figure 5:**
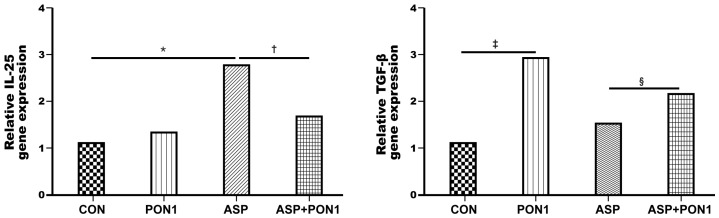
The effect of paraoxonase-1 (PON1) on Th2-mediated inflammation. The gene expression of IL-25 was significantly increased after the treatment of the Aspergillus protease (ASP) antigen but decreased after PON1 treatment in mouse lung epithelial cells. However, PON1 treatment significantly increased the expression of TGF-β in the primary lung epithelial cells. Data are expressed as the mean ± SD of four independent experiments, each performed in triplicate. * *p *= 0.002; † *p *= 0.006; ‡ *p* = 0.004; § *p* = 0.009.

After the treatment of the *Aspergillus* protease antigen and PON1 in the MLE-12 cells, the gene expression levels of IL-25 and TGF-β significantly increased (*p *= 0.002 and *p *= 0.004, respectively). However, PON1 treatment significantly decreased the expression of IL-25 (*p *= 0.006) and considerably increased TGF-β (*p *= 0.009) in the MLE-12 cells pretreated with *Aspergillus* protease (Figure 5).

### 2.6. Activation and Maturation of Dendritic Cells

**Figure 6:**
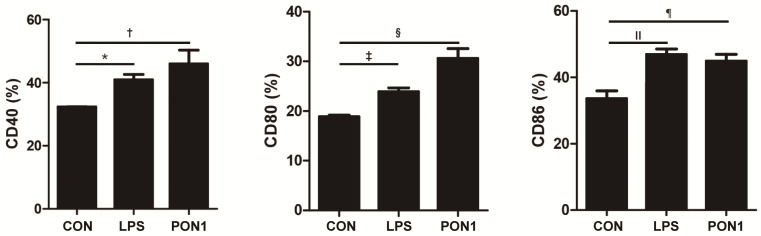
The effect of paraoxonase-1 (PON1) on dendritic cell maturation. Lipopolysaccharide (LPS) and PON1 treatment significantly increased the expressions of CD40, CD80, and CD86 in immature dendritic cells. Data are expressed as the mean ± SD of four independent experiments, each performed in triplicate. * *p *= 0.036; † *p *= 0.003; ‡ *p* = 0.028; § *p* = 0.004; ǁ *p* = 0.035; ¶ *p* = 0.039.

The expression of CD40 (*p* = 0.036), CD80 (*p* = 0.028), and CD86 (*p* = 0.035) was significantly increased after activation with LPS. Moreover, PON1 treatment significantly increased the expression of costimulatory molecules such as CD40 (*p* = 0.003), CD80 (*p* = 0.004), and CD 86 (*p* = 0.039) in immature DCs (Figure 6).

## 3. Discussion

Asthma is a chronic inflammatory airway disease involving multiple cells and cellular components characterized by Th2-mediated eosinophilic inflammation, mucus hypersecretion, and AHR [20]. Recent studies have demonstrated that the immune system and gut microbiota are closely associated with the balance of Th17 cells and Tregs, which possess opposing functions, and that vitamin D plays a critical role in the process [21,22]. Current therapies for asthma depend on the suppression of nonspecific immune responses with corticosteroids or the inflammatory mediator antagonist [23]. Several monoclonal antibodies have been introduced and approved for the management of severe asthma. IgE-targeting omalizumab was the first biologic therapy developed to specifically treat allergic asthma in 2003 [24]. From 2015 to 2017, IL-5/IL-5-receptor therapies, including mepolizumab, reslizumab, and benralizumab, were approved for severe eosinophilic asthma [25]. Broader approaches targeting the IL-4/13 receptor with dupilumab or thymic stromal lymphoprotein were approved in those with difficult-to-treat asthma [26].

MSC-derived EVs have been reported as promising candidates for treating allergic airway diseases as they can modulate immune function [27,28]. Previous studies have shown that ASC-derived EVs ameliorate allergic airway inflammation through the suppression of Th2 cytokine production and the induction of Treg expansion [8,10,29]. Furthermore, ASC-derived EVs have been shown to reduce AHR and collagen fiber deposition in lung parenchyma in asthmatic mice [8]. ASC-derived EVs are the main paracrine effector of ASCs. They play a crucial role in intracellular communication by transferring important biomolecules [30]. EVs exert their biological functions by delivering various cellular components, such as proteins, mRNAs, and microRNAs, to recipient cells [31]. Recently, our study reported that gene expression levels of PON1 remarkably decreased in the lung tissue of asthmatic mice, but that the expression of PON1 significantly increased after treatment with ASC-derived EVs [9]. The microRNAs and pulmonary genes, such as PON1, Bex2, Igfbp6, and Scgb1c1, by ASC-derived EVs induce the expansion of Tregs secreting IL-10 and TGF-β, which leads to a decrease in allergy-specific Th2 cytokines (IL-4, IL-5, and IL-13), pulmonary eosinophil infiltration, allergy-specific IgG1 and IgE production, and AHR in asthmatic mice [9,32]. These results suggest that PON1 may be involved in the immune suppression mechanisms of ASC-derived EVs in allergic airway diseases. To our knowledge, this is the first study to investigate the potential role of PON1 in the modulation of allergic airway inflammation through ASC-derived EVs.

The PON gene family includes three known members located adjacent to each other on chromosome 7. PON1 is a 355 amino acid, 43 kDa glycoprotein that is expressed in the liver and secreted into the bloodstream with high-density lipoproteins (HDLs) [33,34]. PON2 and PON3 are intracellular enzymes, and they play roles in the mitochondria and plasma membrane [35,36]. However, PON1 remains the most popular member of this family. PON1 is known for preventing atherosclerosis through lipid-modifying properties, anti-oxidant activity, anti-inflammatory, anti-thrombosis, and anti-apoptosis effects [37]. Several PON1 gene polymorphisms may affect PON1 activity, particularly Q192R and L55 polymorphisms [38]. Decreased PON1 activity has been associated with the development of several diseases, such as coronary artery disease, lung cancer, obstructive sleep apnea, and chronic obstructive pulmonary disease [39,40,41,42]. Recent studies showed that decreased PON1 activity may be involved in the pathogenesis and severity of asthma, suggesting that PON1 can affect the occurrence and development of asthma by reducing inflammation [11,43,44].

The present study has shown that the intraperitoneal and intranasal administration of PON1 to asthmatic mice results in a significant reduction in allergic airway inflammation and the remarkable alleviation of AHR. PON1 treatment significantly decreased the total inflammatory cells and eosinophils in the BALF and improved eosinophilic lung inflammation. Along with the increased expression of PON1 in lung tissue by ASC-derived EVs, these findings support the fact that PON1 with anti-oxidant properties suppressed the recruitment of eosinophils to the lung and improved AHR in asthmatic mice. This is consistent with the results of previous reports using the ASC supernatant or ASC-derived EVs [8,21]. In addition, treatment with intraperitoneal and intranasal PON1 significantly lowered the total and OVA-specific IgE and the total IgG1 levels. The levels of IL-4, IL-5, and IL-13 were significantly decreased in the BALF and LLNs, whereas IFN-γ and TGF-β were significantly increased in the BALF following intraperitoneal and intranasal PON1 treatment. Consistently, our study also highlights that PON1 inhibits Th2-mediated inflammation via the downregulation of Th2 cytokines and upregulation of regulatory and Th1 cytokines production.

IL-25 has been associated with Th2-mediated inflammation and identified as one of the initiating cytokines of the Th2 allergic responses [45]. In the present study, the expression of IL-25 in the MLE cells was significantly increased as a result of immune responses to the *Aspergillus* protease antigen, but this elevation was remarkably decreased by PON1 treatment. Furthermore, PON1 treatment marked elevated anti-inflammatory cytokines, TGF-β, in the MLE cells stimulated with *Aspergillus* protease. Mature myeloid DCs express large amounts of co-stimulatory molecules, such as CD40, CD80, and CD86, that are prerequisites for their potent T cell sensitizing capacity [46]. Our study showed that the expression of costimulatory molecules such as CD40, CD80, and CD86 in immature DCs is significantly increased after PON1 treatment. Taken together with the immunomodulatory mechanisms of allergic airway diseases by ASCs themselves or the ASC-derived EVs, these findings indicate that PON1 plays a pivotal role in suppressing allergic airway inflammation by ASC-derived EVs.

This study has some limitations. To clarify these findings and apply them to clinical settings, it is essential to conduct research on the immunomodulatory effects of PON1 on allergic airway inflammation in asthmatic patients. Furthermore, future studies need to address the exact receptors and signaling cascade accountable for the immunomodulatory mechanisms of PON1 in allergic airway diseases.

## 4. Materials and Methods

### 4.1. Animals

Five-week-old female C57BL/6 mice were purchased from Samtako Co. (Osan, Republic of Korea, http://www.samtako.co.kr) and bred in specific pathogen-free animal facilities during the experiments. Female mice were used because they are more sensitive to the development of allergic airway inflammation and AHR than male mice after the OVA challenge [47]. The protocol for the animal study was approved by the Institutional Animal Care and Use Committee of the Pusan National University School of Medicine (Approval No. PNU-2023-0313).

### 4.2. Mouse Model of Allergic Airway Inflammation

**Figure 7:**
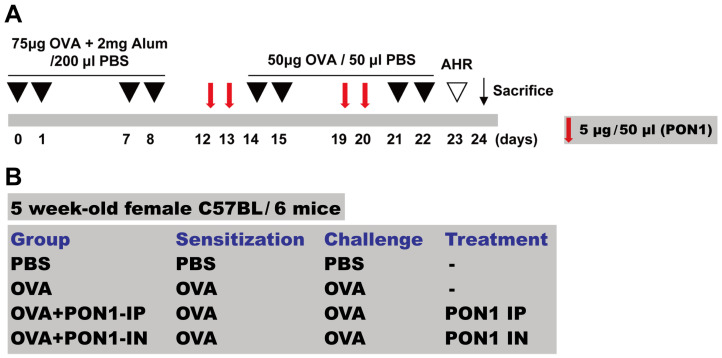
The experimental protocol. (**A**) The mice were sensitized on days 0, 1, 7, and 8 by the intraperitoneal injection of ovalbumin (OVA) and challenged intranasally on days 14, 15, 21, and 22 with OVA. On days 12, 13, 19, and 20, 5 μg/50 μL of the paraoxonase-1 (PON1) were administered intraperitoneally (IP) or intranasally (IN) (red arrow). Airway hyperresponsiveness (AHR) was assessed on day 23 (white arrowhead), and the mice were euthanized with CO_2_ after 2 days from the last challenge (black arrow). (**B**) The mice were divided into four groups according to the different sensitization, challenge, and treatment methods.

We induced a mouse model of allergic airway inflammation according to the previous protocols with minor modifications [5,8]. In short, sensitization was conducted by injecting 75 μg of OVA intraperitoneally (Sigma-Aldrich, St. Louis, MO, USA, http://www.sigmaaldrich.com) with 2 mg of aluminum hydroxide (Sigma-Aldrich) in 200 μL of phosphate-buffered saline (PBS) on days 0, 1, 7, and 8. On days 14, 15, 21, and 22 after the initial sensitization, the mice were challenged by the intranasal instillation of 50 μg of OVA in 50 μL of PBS. On day 24, every subject was sacrificed (Figure 7A).

### 4.3. Intraperitoneal and Intranasal Administration of PON1

Recombinant mouse serum paraoxnase/arylesterase 1 (# CSB-YP347323MO) was purchased from Cusabio Technology LLC (Houston, TX, USA). The molecular weight of the PON1 protein was 40 kDa. The PON1 glycoprotein is composed of 354 amino acids. To evaluate the effect of PON1, the intranasal and intraperitoneal injections of PON1 (5 μg/50 μL) were performed on days 12, 13, 19, and 20. Mice were divided into four groups, with five mice per group: (a) the PBS group was sensitized, pretreated, and challenged with PBS; (b) the OVA group was sensitized with OVA, pretreated with PBS, and then challenged with OVA; (c) the OVA+PON1-IP group was sensitized with OVA, intraperitoneally pretreated with PON1, and then challenged with OVA; and (d) the OVA+PON1-IN group was sensitized with OVA, intranasally pretreated with PON1, and then challenged with OVA (Figure 7B).

### 4.4. Measurement of AHR to Methacholine

As described in previous studies, noninvasive whole-body plethysmography (Allmedicus, Seoul, Republic of Korea) was performed 24 h after the last challenge to assess AHR in conscious, unstrained mice [5,8]. We put the mice in the plethysmography chamber and exposed them to increasing concentrations of aerosolized methacholine at 0, 12.5, 25, and 50 mg/mL for 10 min. The enhanced pause (Penh) was measured automatically based on the mean pressure generated in the chamber during inspiration and expiration, combined with the time of each phase. The average of the Penh values calculated during each 3 min interval was used.

### 4.5. Differential Cell Counting in Bronchoalveolar Lavage Fluid

After sacrificing the mice, the bronchoalveolar lavage fluid (BALF) was collected as described in our previous studies [5,8,29,48]. The samples underwent centrifugation at 1500 rpm for 5 min at 4 °C. The supernatants from the BALF were frozen immediately at −70 °C. We resuspended the cell pellets and washed them in PBS. The total cell numbers were counted by a hematocytometer. We stained the BALF cell smears with a Diff-Quik solution (Sysmex Co., Kobe, Japan) to determine the differential cell counts on the basis of the conventional morphological criteria. The examination for at least 500 cells per slide was conducted to obtain the differential leukocyte counts.

### 4.6. Lung Histology and Inflammation Scoring

The lung tissue was removed after lavage. It was fixed in 10% neutral formalin for 36 h and then embedded in paraffin. The sections were stained with hematoxylin and eosin (H&E) to identify eosinophils. The index of lung inflammation was assessed by scoring the inflammatory cell infiltrates around airways for the greatest severity (0, normal; 1, ≤3 cells thick; 2, 4–10 cells thick; 3, ≥10 cells thick) and their overall extent (0, normal; 1, <25% of sample; 2, 25–50% of sample; 3, 51–75% of sample; 4, ≥75% of sample) [5,8,29,48]. The score was calculated by multiplying the severity by the extent. Furthermore, periodic acid Schiff (PAS) staining was performed to identify mucin-secreting cells.

### 4.7. Measurement of Serum Immunoglobulin

Mice were euthanized with CO_2_ 48 h after the last challenge. As a post-mortem procedure, the serum was collected via the cardiac puncture of mice. Total and OVA-specific immunoglobulins (IgE, IgG1, IgG2a) were evaluated by the enzyme-linked immunosorbent assay (ELISA) according to the manufacturer’s instructions (R&D Systems, Minneapolis, MN, USA). Absorbance at 450 nm was measured using an ELISA plate reader (Molecular Devices, Sunnyvale, CA, USA).

### 4.8. Expression of Cytokines in the BALF and Lung Draining Lymph Nodes

Lung draining lymph nodes (LLNs) were harvested between the trachea and both lung lobes of asthmatic mice. The cells were isolated from the LLNs as previously reported [5,8,29,48] and plated in a 48-well coated with 0.5 μg/mL of the CD3 antibody (BD Pharmingen^TM^, BD Bioscience, San Jose, CA, USA) at a concentration of 10^6^ cells/mL in Roswell Park Memorial Institute (RPMI) 1640 with 10% fetal bovine serum (FBS). The plated cells were incubated for 72 h at 37 °C with 5% CO_2_. After stimulation, the concentrations of interleukin (IL)-4, IL-5, IL-13, interferon (IFN)-γ, and transforming growth factor (TGF)-β in the BALF and in the LLN supernatants were measured using ELISA kits according to the manufacturer’s instructions (eBioscience, San Diego, CA, USA). The absorbance of the final reactant was determined at 450 nm with an ELISA plate reader (Molecular Devices, Sunnyvale, CA, USA).

### 4.9. Treatment of Lung Epithelial Cells and Analysis of Gene Expression

Mouse lung epithelial cells (MLE-12 cells) were purchased from ATCC (Manassas, VA, USA). In total, 4 × 10^5^ MLE cells were placed in a 24-well and incubated overnight at 37 °C with 5% CO_2_. The cells were left untreated (as a control) and pretreated with 200 ng/mL of *Aspergillus* protease antigen for 2 h, and then they were treated with 1 μg/mL of PON1 for 2 h. The cells and culture supernatants were acquired and kept at −80 °C for quantitative real-time polymerase chain reaction (qRT-PCR). Total RNA from MLE-12 cells was extracted using 1 mL QIAzol (Qiagen science, Valencia, CA, USA), transcribing 2 μg of RNA using the reverse transcriptase of the Moloney murine leukemia virus. (Promega, Madison, WI, USA). IL-25 (forward, 5′-TGGCAATGATCGTGGGAACC-3′; reverse, 5′-GAGAGATGGCCCTGCTGTTGA-3′) and TGF-β (forward, 5′-GCCATCTATGAGAAAACCAAAG-3′; reverse, 5′-TAGTTCACACCTCGTTGTAC-3′) RNA levels were quantified, relative to the housekeeping gene GAPDH (forward, 5′-TACCCCCAATG TGTCCGTC-3′; reverse, 5′-AAGAGTGGGAGTTGCTGTTGAAG-3′), using a LightCycler 96 Real-Time PCR system (Roche, Basel, Switzerland) in accordance with the manufacturer’s instructions. The relative gene expression level was calculated by the comparative Ct (2^−ΔΔCt^) method.

### 4.10. In Vitro Dendritic Cells Stimulation Assay

Bone marrow-derived dendritic cells (BMDCs) were differentiated from bone marrow cells following the previously reported method with minor modification [49]. In short, the femurs and tibias of 7-week-old C57BL/6 mice were the source of bone marrow cells. The bone marrow cells were collected, washed, and cultured in a complete RPMI 1640 medium. The medium contained 10% heat-inactivated FBS, 2 mM glutamine, 50 μM 2-mercaptoethanol, 100 μg/mL streptomycin, 100 unit/mL penicillin (Invitrogen, Carlsbad, CA, USA), 10 ng/mL recombinant mouse granulocyte–macrophage colony-stimulating factor (GM-CSF) and 10 ng/mL recombinant mouse IL-4 (R&D Systems, Minneapolis, MN, USA). Granulocytes that were non-adherent were removed after 24 h of culture. A fresh, complete medium was supplemented every other day. All cultures were incubated at 37 °C with 5% CO_2_. After culture for 7 days, characteristic CD-specific markers (CD11c+) were expressed in 80% of the cells, as determined by flow cytometry. Immature BMDCs were cultivated in 6-well plates at 4 × 10^6^ cells/well in 3 mL of complete RPMI 1640 medium enriched with 10% FBS and 40 ng/mL GM-CSF. BMDCs were left untreated (as a control) or were stimulated with either 1 mg/mL of LPS or 1 μg/mL of EV for 24 h. Either LPS- or PON1-stimulated BMDCs were then incubated with the anti-mouse antibodies as follows: FITC-conjugated anti-CD11c and PE-conjugated anti-CD40, -CD80, and -CD86 (eBioscience, San Diego, CA, USA). Flow cytometry was conducted utilizing a FACS Canto II cytometer (BD Biosciences, San Jose, CA, USA).

### 4.11. Statistical Analysis

All procedures were performed at least three times. Data are expressed as means ± standard deviations. Statistical significance was determined by Student’s *t*-test using GraphPad Prism 5.0 software (GraphPad Software Inc., La Jolla, CA, USA). A value of *p* < 0.05 was considered significant.

## 5. Conclusions

The intraperitoneal and intranasal administration of PON1 significantly reduced allergic airway inflammation and improved AHR through the downregulation of IL-4, IL-5, and IL-13 and upregulation of TGF-β in asthmatic mice. Furthermore, PON1 treatment marked decreased Th2-mediated inflammation through the activation of dendritic cells, accompanied by a decrease in IL-25 and an increase in TGF-β in MLE-12 cells.

## Data Availability

The data used to support the findings of this study are available from the corresponding author upon request.
